# New In Vitro Coculture Model for Evaluating Intestinal Absorption of Different Lipid Nanocapsules

**DOI:** 10.3390/pharmaceutics13050595

**Published:** 2021-04-21

**Authors:** Norraseth Kaeokhamloed, Emillie Roger, Jérôme Béjaud, Nolwenn Lautram, Florence Manero, Rodolphe Perrot, Marie Briet, Chadi Abbara, Samuel Legeay

**Affiliations:** 1 MINT, INSERM U1066, CNRS UMR 6021, SFR ICAT, University of Angers, F-49000 Angers, France; norraseth.kaeokhamloed@univ-angers.fr (N.K.); emilie.roger@univ-angers.fr (E.R.); jerome.bejaud@univ-angers.fr (J.B.); nolwenn.lautram@univ-angers.fr (N.L.); 2 SCIAM, SFR ICAT, University of Angers, F-49000 Angers, France; florence.manero@univ-angers.fr (F.M.); rodolphe.perrot@univ-angers.fr (R.P.); 3 Laboratoire de Pharmacologie-Toxicologie, CRPV, Angers University Hospital Center, F-49000 Angers, France; chadi.abbara@chu-angers.fr (C.A.); marie.briet@chu-angers.fr (M.B.)

**Keywords:** intestinal absorption, Caco-2, HMEC-1, apparent permeability, lipid nanocapsule, förster resonance energy transfer

## Abstract

Standard models used for evaluating the absorption of nanoparticles like Caco-2 ignore the presence of vascular endothelium, which is a part of the intestinal multi-layered barrier structure. Therefore, a coculture between the Caco-2 epithelium and HMEC-1 (Human Microvascular Endothelial Cell type 1) on a Transwell^®^ insert has been developed. The model has been validated for (a) membrane morphology by transmission electron microscope (TEM); (b) ZO-1 and β-catenin expression by immunoassay; (c) membrane integrity by trans-epithelial electrical resistance (TEER) measurement; and (d) apparent permeability of drugs from different biopharmaceutical classification system (BCS) classes. Lipid nanocapsules (LNCs) were formulated with different sizes (55 and 85 nm) and surface modifications (DSPE-mPEG (2000) and stearylamine). Nanocapsule integrity and particle concentration were monitored using the Förster resonance energy transfer (FRET) technique. The result showed that surface modification by DSPE-mPEG (2000) increased the absorption of 55-nm LNCs in the coculture model but not in the Caco-2. Summarily, the coculture model was validated as a tool for evaluating the intestinal absorption of drugs and nanoparticles. The new coculture model has a different LNCs absorption mechanism suggesting the importance of intestinal endothelium and reveals that the surface modification of LNCs can modify the in vitro oral absorption.

## 1. Introduction

Drug administration by the oral route is considered the most accepted one by patients for drug delivery due to its convenience. However, some drugs present a low oral bioavailability due to low drug solubility or low intestinal permeability. This low permeability can be explained by the intestine’s complex multilayer structure, consisting of the mucus barrier, the enterocytic barrier, and the endothelial barrier [1,2,3]. To improve oral drug delivery, drug encapsulation into nanocarriers, such as lipid nanocapsules (LNCs), is currently one of the most promising technologies. LNCs consist of an oily core enclosed by a shell of pegylated surfactant and phosphatidylcholine and can be prepared by a well-known low-energy emulsification process: The phase-inversion temperature method [4,5]. LNCs have sizes ranging from 20 to 100 nm and can also be prepared with different surface-chemistry. For example, anionic LNCs can be produced by the addition of DSPE-mPEG (2000) [6] and cationic LNCs by adding stearylamine [7] or chitosan [8,9]. Previous studies demonstrated that LNCs enhance the in vivo oral bioavailability of paclitaxel, fondaparinux, albendazole, and praziquantel; and the in vitro intestinal absorption of paclitaxel, Sn38, decitabine, acyclovir, and efavirenz [7,10,11,12,13,14,15,16]. In addition, the use of the in vitro Caco-2 model allowed us to describe that LNCs are mainly transported via active endocytosis, or more precisely, through clathrin-dependent and caveolae-dependent transport mechanisms. Recently, the application of the Förster resonance energy transfer (FRET) technique coupled with Nanoparticle Tracking Analysis (NTA) demonstrated that few LNCs (around 0.3% of the initial quantity of LNCs) were able to be transported intact by transcytosis after passage through Caco-2 cell monolayer [17].

FRET is a useful technique to monitor the integrity of nanoparticles. It is based on the interaction between two spatially closed (1–10 nm) fluorophores in which the emission spectrum of the FRET-donor overlaps with the excitation spectrum of the FRET-acceptor. The efficacy of the energy transfer between the two fluorophores is related to their proximity [18,19,20]. Thus, the loss of FRET-nanoparticles integrity will cause the FRET fluorophores to be released into the external medium and disperse, widening the fluorophores’ proximity and resulting in the disappearance of the FRET-acceptor emission spectrum. As such, FRET is currently the only technique that can be used to follow the passage of intact nanoparticles in biological fluids or organisms. By a combination of the FRET technique with the NTA, Roger et al. developed a quantitative method to measure the particle concentration of intact LNCs [21], making this a precise tool for monitoring the membrane transport of nanocarriers.

Nevertheless, to date, the study on the membrane transport of LNCs across the intestinal barrier has been done only in the Caco-2 model, which consists of a monolayer of immortalized enterocyte cells cultivated on a semi-permeable membrane [22,23,24,25]. This model is the most commonly used and considered as a reference model for evaluating intestinal drug permeability. The Caco-2 model is used by regulatory agencies such as the USFDA (U.S. Food and Drug Administration) and the ICH (International Council for Harmonization) to establish the biopharmaceutics classification system (BCS) that classifies drugs based on permeability and solubility. The BCS classes can be defined by the apparent permeability (P_app_) of drugs in the Caco-2 model. Drugs with high permeability (class I and II) and low permeability (class III and IV) are defined by the P_app_ > 10 × 10^−6^ cm/s and P_app_ < 2 × 10^−6^ cm/s, respectively. Drugs with the P_app_ between 2–10 × 10^−6^ cm/s are classified on a case-by-case basis because other pharmacokinetics parameters can influence their permeability [26,27,28]. Over the past few years, the Caco-2 model has been improved with other cell types added as a coculture system (e.g., HT-29, Raji-B coculture model) [22,29,30]. However, all these models for studying intestinal absorption ignore the endothelium layer that LNCs have to cross before reaching blood circulation. A recent study demonstrated that the intestinal endothelium plays a major role as a barrier against antigen and nutrients transport similar to the blood–brain barrier [31]. Another in vivo study in rats suggested that the disruption of the endothelium allows pathogens to enter the systemic circulation, strengthening the involvement of the intestinal endothelium in the mechanisms of oral absorption [32,33]. However, the role of intestinal endothelium in regulating drug absorption has never been studied. Besides, endothelium in other barriers such as the pulmonary endothelium (air–blood barrier) has been recently found to have a significant role in regulating the absorption of drugs and macromolecules [34]. Therefore, the role of the intestinal endothelium on drugs and nanoparticle absorption needs to be elucidated. Recently, Kasper et al. [35] developed an in vitro intestinal coculture model comprising Caco-2 and human hemangiosarcoma-derived endothelial cells (ISO-HAS-1), but the model was used for studying the pathophysiology of the inflamed intestinal membrane, not for drug absorption. The objective of the present study is to develop and validate a new in vitro coculture model in order to systematically evaluate, for the first time, the transport of different LNCs across the intestinal epithelial-endothelial barrier.

In this context, we developed a new coculture model that can better mimic the gut–blood barrier structure for studying the membrane transport of intact lipid nanocapsules. Caco-2 cells and Human Microvascular Endothelial Cell type 1 (HMEC-1) were seeded on the apical and basolateral side of the Transwell^®^ plate, respectively. This model was characterized by membrane morphology, tight junction expression, and trans-epithelial electric resistance. Five different drugs were selected based on their physicochemical characteristics as a representation of drugs in general with different permeability and solubility according to the BCS [26,27,28]. Their permeability was evaluated in the coculture model in comparison with the reference Caco-2 model for the conformity with BCS. LNCs with different sizes (55 and 85 nm) and different surface chemistries (DSPE-mPEG (2000) and stearylamine) were formulated and loaded with FRET dyes [35]. The transport of intact FRET-LNCs was investigated across the newly developed coculture compared to the well-established Caco-2 model, using the quantitative FRET fluorimetry technique coupled with the NTA to quantify the particle concentration of intact LNCs.

## 2. Materials and Methods

### 2.1. Materials

Caco-2 and HMEC-1 cells were obtained from American Type Culture Collection (Manassas, VA, USA). Sodium chloride, sodium tetraphenylborate, ethyl acetate, dichloromethane, methanol, triton X-100, Trizma^®^ (base), anti-β-catenin rabbit mAb, anti-TJP1 (tight junction protein-1; a.k.a. ZO-1, zonula occludens-1) rabbit mAb, collagen type 1 (calf skin), 4′,6-diamidino-2-phenylindole (DAPI), Dulbecco’s modified Eagle’s medium (DMEM D6429, with 4500 mg/L glucose, l-glutamine, sodium pyruvate, and sodium bicarbonate), dimethyl sulfoxide (DMSO), epidermal growth factor, hydrocortisone HCl powder for injection, Osmium tetroxide (OsO_4_), Epon™ 812 resin, metoprolol tartrate, propranolol HCl, naproxen, atenolol and furosemide, and stearylamine were purchased from Sigma-Aldrich (Saint-Quentin Fallavier, France). Hank’s balanced salted solution (HBSS), MCDB 131 medium (Gibco 10372-019), penicillin-streptomycin solution, goat anti-rabbit IgG Alexa Flour^®^ 488, DiI (1,10-dioctadecyl-3,3,30,30-tetramethyl-indocarbocyanine perchlorate), and DiD (1,1′-dioctadecyl-3,3,3′,3′-tetramethylindo-dicarbocyanine perchlorate) were purchased from Thermofisher (Villebon-sur-Yvette, France). Amphotericin B, Phosphate-buffered saline (PBS), and l-glutamine were purchased from PAA Laboratories (Toronto, ON, Canada). Fetal bovine serum (FBS) was purchased from Biowest (Nuaillé, France). Paraformaldehyde 32% and glutaraldehyde 25% were purchased from Electron Microscopy Science (Hatfield, PA, USA). Nonessential amino acid (NEAA) was purchased from Lonza (Verviers, Belgium). Ultrapure water was obtained from a Milli-Q^®^ Advantage A10 System (Merck Millipore, Darmstadt, Germany). Costar^®^ Transwell^®^ (12-well, polycarbonate membrane filters, 0.4 µm pore size, 1.12 cm^2^ growth area) and T75 cell culture flasks were purchased from Costar (New York, NY, USA). Captex^®^ 8000 (glyceryl tricaprylate) was kindly provided by Abitec Corporation (Columbus, OH, USA). Lipoid^®^ S75-3 (phosphatidylcholine and phosphatidylethanolamine mixture) was purchased from Lipoid GmbH (Steinhausen, Switzerland); Kolliphor^®^ HS-15 (PEG 660 and polyethylene glycol 660 hydroxystearate mixture) from BASF (Ludwigshafen, Germany); and DSPE-mPEG(2000) (1,2-distearoyl-sn-glycero-3-phosphoethanolamine-*N*-[methoxy(polyethylene glycol)-2000] (ammonium salt)) from Avanti Polar Lipids (Alabaster, AL, USA).

### 2.2. Caco-2 and HMEC-1 Cells Culture

Caco-2 cells were cultured between passage 18 and 27 in DMEM medium supplemented with 20% *v*/*v* FBS, 1% *v*/*v* non-essential amino acids and 100 UI/mL penicillin, 0.5 mg/mL streptomycin. HMEC-1 cells were cultured between passage 4 and 12 in MCDB 131 medium supplemented with 10% *v*/*v* FBS, 2 mM/mL l-glutamine, 100 UI/mL penicillin, 0.5 mg/mL streptomycin, 2.5 µg/mL amphotericin B, 1 µg/mL hydrocortisone, and 0.01 µg/mL epidermal growth factor. Cells were cultured in a T75 flask (75 cm^2^) and incubated at 37 °C in humidified air with 5% CO_2_.

### 2.3. Caco-2/HMEC-1 Monoculture and Coculture on Transwell^®^

The polycarbonate membrane filters (0.4 µm pore size, 1.12 cm^2^ growth area) in Transwell^®^ inserts were coated with collagen type-1 for 8 µg/cm^2^ on the apical side and 25 µg/cm^2^ on the basolateral side [28]. Caco-2 cells (1 × 10^5^ cells) were seeded onto the apical side of the coated filter and cultured in DMEM medium, which was changed every 2–3 days. On day 18, the Transwell^®^ inserts were taken out, flipped upside down, and submerged under DMEM medium in a sterile basin with no bubbles trapped underneath the filter. Then, 5 × 10^4^ HMEC-1 cells were seeded onto the basolateral surface of the filter and were incubated for 2 h at room temperature. The inserts were then placed back into the Transwell^®^ chambers. The Caco-2 media and the HMEC-1 media were filled in the upper chamber and the lower chamber, respectively, and were changed every two days. The coculture membranes were used on day 22 of Caco-2 cells and day 4 of HMEC-1 cells. Moreover, 1 × 10^5^ Caco-2 cells and 5 × 10^4^ HMEC-1 cells were separately seeded on the Transwell^®^ inserts for the monoculture and then used as control.

### 2.4. Membrane Morphology

#### 2.4.1. Transmission Electron Microscopy (TEM)

Caco-2/HMEC-1 cell membranes in Transwell^®^ inserts were washed with HBSS and fixed with 2.5% *v*/*v* glutaraldehyde in phosphate buffer pH 7.4 for 1 h at room temperature, then replaced with phosphate buffer pH 7.4. Afterward, 1% *w*/*v* OsO_4_ was added to the cell samples and kept for 1 h at room temperature. Then, the samples were washed three times by deionized water and subsequently dehydrated by 50%, 70%, and 95% ethanol twice and four times with 100% ethanol. Next, the samples were embedded in Epon™ 812 resin, which was left to polymerize for 24 h at 60 °C. Thin slices (60 nm) were cut from each sample using Leica UC7 ultramicrotome (Leica microsystems, Wetzlar, Germany) and deposited onto copper grids. The samples were stained with 3% uranyl acetate in 50% ethanol for 5 min and washed with deionized water. The samples were left to dry and then examined using the JEOL JEM-1400 electron microscope (JEOL, Tokyo, Japan).

#### 2.4.2. Confocal Fluorescence Microscopy

Caco-2, HMEC-1, and Caco-2/HMEC-1 cell layers on Transwell^®^ inserts were fixed for 20 min with 4% *v*/*v* paraformaldehyde at room temperature. The cells were washed three times with TBS and then permeabilized for 10 min with 0.5% *v*/*v* Triton X-100 in TBS at room temperature. After washing out three times with TBS, 5% *w*/*v*, FBS was added and rinsed out three times by TBS after 1 h. Anti-β-catenin rabbit monoclonal antibodies (mAb) (1:300 in 2% *w*/*v* FBS in TBS) and Anti-ZO-1 rabbit mAb (1:300 in 2% *w*/*v* FBS in TBS) were separately added to the cells kept overnight at 4 °C. Then cells were rinsed out three times by TBS, and the fluorescence-labeled secondary antibody goat anti-rabbit IgG Alexa Fluor^®^ 488 (1:500 in 2% *w*/*v* FBS in TBS) was added and kept overnight at 4 °C. Then, the cells were washed three times with TBS before stained with DAPI (3 µg/mL in TBS) for 10 min at room temperature. Finally, the filter membranes were cut from the Transwell^®^ and mounted between microslides. The immunofluorescent staining images of cell confluency and tight junction structure were characterized by Leica TCS SP8 laser-scanning confocal microscope (Leica Microsystems, Heidelberg, Germany) with the excitation and emission wavelength of 488 and 520 nm, respectively, for tight junction protein ZO-1 and adherens junction protein β-catenin, and 405 nm and 461 nm, respectively, for cell nuclei. The software Leica Application Suite X was used for 3D visualization.

#### 2.4.3. Trans-Epithelial Electrical Resistance (TEER)

TEER (Ω·cm^2^) of the Caco-2 and the coculture cell layers were measured by the Millicell^®^ ESR-2 volt-ohmmeter (Merck Millipore Corporation, Burlington, MA, USA) on days 4, 11, 18, 21, and 22. The values were corrected by the resistance of blank Transwell^®^ insert following the equation:

TEER = (R_total_ − R_blank_) × A (1)

when R_total_ is the measured resistance (Ω), R_blank_ is the arithmetic mean of the resistance of blank Transwell^®^ insert (110 Ω), and A is the area of Transwell^®^ filter (1.12 cm^2^).

### 2.5. Transport Assay of the Free Drugs

#### 2.5.1. Transport Assay Experiment

Five reference drugs were chosen as a representation of drugs with different solubility and permeability according to the biopharmaceutical classification system (BCS) [36] (see Table 1). The drugs were firstly dissolved in ultrapure water (if necessary, methanol could be used to dissolve the drugs with the final concentration of methanol less than 0.02% *v*/*v*) and then serially diluted in HBSS to 5 µM. 1.5 mL of HBSS and 0.5 mL of diluted drug solutions were added to the basolateral and apical chambers of the Transwell^®^ wells, respectively. Studies were performed on the Caco-2/HMEC-1 coculture model, the Caco-2 model, the HMEC-1 model, and Transwell^®^ without cells (control). The plates were incubated for 2 h at 37 °C with humidified air and 5% CO_2._ Afterward, the apical and the basolateral media were collected and analyzed by HPLC-UV.

#### 2.5.2. Drug Analysis by HPLC-UV

HPLC analysis was performed using the Agilent 1200 HPLC system (Agilent Technologies, Les Ulis, France) with a UV detector (deuterium lamp light source) and with the Uptisphere^®^ C18-ODB 100 × 2.1 mm, 5 µm column (Agilent Technologies, Les Ulis, France). Sample preparation was explained in Appendix A. The analysis run time was 20 min. The mobile phase consisted of phase A (phosphate buffer pH 7.4) and phase B (acetonitrile). In initial conditions, the mobile-phase composition was 15% B; a linear gradient was applied to reach a composition of 80% B after 16 min, maintained 2 min, and then set to return to initial. The flow rate was 0.4 mL/min. Each drug was analyzed separately, and their retention times were: 1.7 min for atenolol, 4.6 min for metoprolol, 7.4 min for propranolol, 6.2 min naproxen, and 5.6 min for furosemide. Quantification was achieved using calibration curves (area ratio with internal standard vs. nominal analyte concentration) fitted by linear least squares regression. The lower limit of quantification (LLOQ) was validated at 50 ng/mL for all substances, and the limit of detection (LOD) was 10 ng/mL.

#### 2.5.3. Apparent Permeability Calculation

The apical-to-basolateral apparent permeability was calculated following the equation:

(2) $$P_{\mathrm{app}}=\frac{\mathrm{dQ}\;}{\mathrm{dt}}\;\times \;\frac{1}{{\mathrm{AC}}_{0}}$$

where dQ/dt is the appearance rate of a drug at the basolateral side (µg/s), *A* is the surface area of the Transwell^®^ filter (1.12 cm^2^), and C_0_ is the initial concentration at the apical side (µg/mL) [21,37].

### 2.6. Formulation of FRET-LNCs

#### 2.6.1. Synthesis of DiI- and DiD-TPB

The fluorescence dyes DiI- and DiD-tetraphenylborate (TPB) were synthesized by the method previously described [4,5,10,21]. Dyes were solubilized in Captex^®^ 8000 at the concentration of 2% *w*/*w*.

#### 2.6.2. Formulation of FRET Lipid Nanocapsules (FRET-LNCs)

Six formulations of FRET-LNCs were prepared based on the phase inversion method [6]. The surface modification was adapted from the ‘one-step (OS) stealth LNCs process’ developed by Lainé et al. [38]. The composition of lipid nanocapsules in each batch is described in Table 2. Firstly, Lipoid^®^ S75-3 was dissolved in the Captex^®^ 8000 containing DiI-TPB and DiD-TPB. Then, Kolliphor^®^ HS-15, purified water, and NaCl were added, as well as the surface modification substances, which were DSPE-mPEG (2000) (anionic) or stearylamine (cationic), if applicable. Under agitation, three heat–cool cycles (60–90 °C) were applied to the mixture. In the last cooling cycle, cold ultrapure water (2 °C) was added at the phase inversion temperature, followed by 5 min of a slow stir. Finally, the suspension of FRET-LNCs was filtrated by a 0.22 µm filter (Minisart^®^) and stored at 2–8 °C.

### 2.7. Characterization of FRET-LNCs

The concentration (particles/mL) and the size distribution of the nanoparticles were determined with the nanoparticle tracking analysis (NTA) technique using a NanoSight NS300 (Malvern Instrument, Worcestershire, UK) with a low volume flow cell and a 450 nm laser. FRET-LNCs suspensions were diluted in ultrapure water by factor 300,000 (*v*/*v*) and then slowly injected into the sample chamber using a 1 mL syringe pump with the rate of 3–4 µL per second. The video sequences of the nanoparticles were captured over 60 s (5 replicates) and then analyzed by NTA analytical software version 3.2.

NTA provides the particle size distribution parameters as D10, D50, and D90; which represents the diameter (nm) at the 10th, 50th (median), and 90th percentiles of the distribution histogram, respectively. Then the span, as a distribution width parameter, is calculated following the equation:

(3) $$\mathrm{Span}=\frac{D90\;-\;D10}{D50}$$

The zeta potential of the FRET-LNCs was determined by laser doppler electrophoresis using Zetasizer^®^ Nano series DTS 1060 (Malvern Instruments SA, Worcestershire, UK).

### 2.8. Transport Assay of Intact LNCs across Membranes

#### 2.8.1. Transport Assay of FRET-LNCs

First, 1.5 mL of HBSS and 0.5 mL of diluted FRET-LNCs (1% *v*/*v* in HBSS) were filled into the basolateral and the apical side of the Transwell^®^ plates, respectively. The plates were incubated for 2 h at 37 °C with humidified air and 5% CO_2_. Then, samples from the basolateral and the apical sides were collected, and fluorescence was analyzed by spectrophotometer. The TEER of all membranes were measured before and after the experiment to ensure their integrity. Membranes with TEER <300 Ω·cm^2^ were excluded from the test.

#### 2.8.2. Quantitative FRET Fluorimetry of Intact LNCs

Fluorescence emission spectra of collected samples were recorded on a FluoroMax^®^ 4 spectrophotometer (Horiba Jobin Yvon Inc., Piscataway, NJ, USA) at room temperature with the 548 nm excitation and 0.5 s integration time. The emission spectra were collected from 555 to 750 nm, with an increment of 1 nm. They were corrected for the lamp source fluctuations and the wavelength-dependent response of the detector. The integrity of nanoparticles was determined by FRET efficiency (proximity ratio) calculated by the following equation:

(4) $$\mathrm{PR}=\frac{A}{A+D}$$

where A and D are the maximum fluorescence intensity of the acceptor (678 nm) and donor (569 nm), respectively. The particle concentration of the nanocarriers was calculated from the standard curve of the FRET acceptor signal. An acceptor signal lower than the limit of detection (LOD = 1382 cps/mA) and/or a PR lower than 0.70 was considered as zero particle concentration.

#### 2.8.3. Transport Efficiency of FRET-LNCs

The transport efficiency (TE) of FRET-LNCs is the percentage of numbers of nanoparticles presenting at the basolateral medium compared to the initial particle concentration at the apical medium. It was calculated by the equation:

(5) $$\mathrm{TE}=\frac{C_{f}V_{B}}{C_{0}V_{A}}\;\times \;100\%$$

where C_f_ is the particle concentration at the basolateral side after 2 h, C_0_ is the initial particle concentration at the apical side (particles/mL), V_A_ is the volume of sample at the apical side (1 mL), and V_B_ is the volume of sample at the basolateral side (1.5 mL).

### 2.9. Statistical Analysis

The experiments were performed at least in triplicate. For statistical comparison, the Kruskal–Wallis test with uncorrected Dunn’s multiple comparison test was the method of statistical analysis. *p*-value of less than or equal to 0.05 was considered statistically significant. All statistical analysis was performed using Prism GraphPad (version 8.4.1, GraphPad Software, San Diego, CA, USA).

## 3. Results and Discussions

### 3.1. Development of the New In Vitro Coculture Model

**Figure 1:**
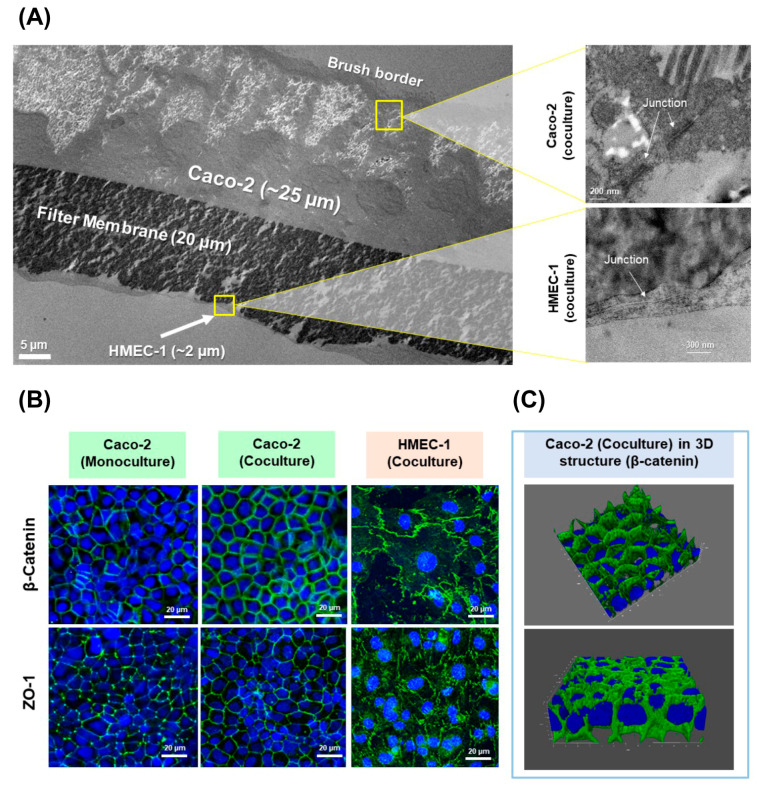
(**A**) Cross-sectioning images of Caco-2 and HMEC-1 coculture layers under TEM (left) and zoomed cross-sectioning images of the junction area between Caco-2 cells (upper right) and HMEC-1 cells (lower right); (**B**) immunofluorescent staining images of Caco-2 monoculture, cocultured Caco-2 and cocultured HMEC-1 layers, characterized by confocal microscopy for the expression of tight junction protein ZO-1 (Alexa Fluor^®^ 488, green) and adherens junction protein β-catenin (Alexa Fluor^®^ 488, green). The images are overlayed with cell nuclei (DAPI, blue); (**C**) the 3D images show only the expression of β-catenin in the cocultured Caco-2 layer and revealed a confluent monolayer with no cell stacking.

The new in vitro 2D coculture model between Caco-2 and HMEC-1 cells was developed and intended as a tool to assess the absorption of drugs and nanoparticles. In order to investigate its morphology, cross-sections of cell layers were observed under TEM (Figure 1A). On day 22, Caco-2 cells exhibited a columnar epithelium monolayer structure with ~25 µm thickness with a brush border (microvilli) on the apical surface. When zooming in between the adjacent borders of two Caco-2 cells, junctional complex structures were observed. The morphology of these Caco-2 cells corresponds to mature human enterocytes [23,39,40] and typical Caco-2 cells described elsewhere [41,42]. Furthermore, the HMEC-1 layer was obtained as a very thin squamous epithelium monolayer with 0.2–2 µm thickness covering the basolateral side of the filter (facing downward). The junctional structure was observed along HMEC-1 cell borders. This morphology of HMEC-1 is closely similar to the structure of human vascular endothelium already described by Young et al. and Ru et al. [40,42]. Thus, with this coculture condition, the morphology of both Caco-2 and HMEC-1 cells was maintained.

In order to investigate the formation of the cell junctional complex, the expression of tight junction protein ZO-1 and adherens junction protein β-catenin were examined by immunofluorescence (Figure 1B). HMEC-1 and Caco-2 cell layers in the coculture model were confluent with the expression of ZO-1 and β-catenin along the cell borders, meaning that tight junctions and adherens junctions were fully developed. A similar structural pattern was observed (Figure 1B) in the Caco-2 monolayer, meaning that the coculture system still maintained the same junctional complex structure as in monoculture. In accordance with our study, Ma et al. described that Caco-2 expressed the ZO-1 as a continuous band along the cell borders after 3 weeks of incubation, while Rüffer et al. found that HMEC-1 expressed ZO-1 and also β-catenin at the cell borders after 3 days of incubation [39]. In addition, a 3D imagery of β-catenin overlayed with cell nuclei (Figure 1C) revealed that the cocultured Caco-2 had a structure of a confluent monolayer with no cell stacking. As such, a confluent monolayer of Caco-2 and HMEC-1 cocultured on the apical and basolateral side of the Transwell^®^ filter, respectively, was obtained.

**Figure 2:**
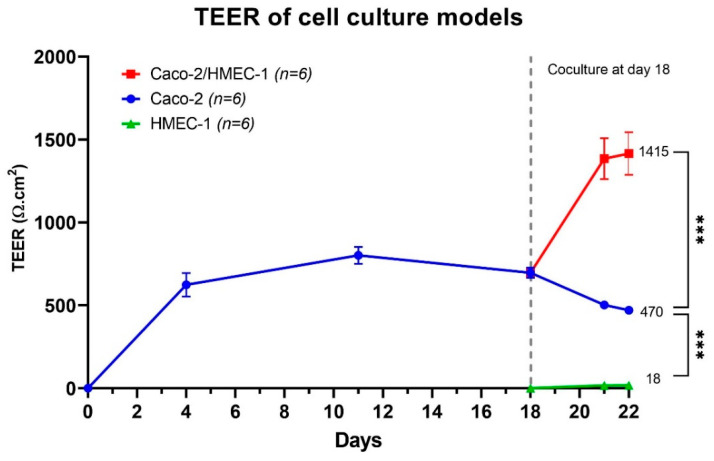
Average TEER (*n* = 6) of Caco-2/HMEC-1 coculture (red), Caco-2 monolayer (blue), and HMEC-1 monolayer (green). The whiskers represent a 95% confidence interval (Kruskal–Wallis: *** *p* ≤ 0.001).

The membrane integrity of cell culture membranes was also monitored by TEER measurement from the beginning to the end of the cell culture. As shown in Figure 2, the average TEER of the Caco-2/HMEC-1 coculture model on day 22 had the highest level, with an average of 1415 ± 331 Ω·cm^2^. This average is significantly (Kruskal–Wallis) higher than that of the Caco-2 model (470 ± 46 Ω·cm^2^), which was also significantly (Kruskal–Wallis) higher than HMEC-1 monolayer (18 ± 6 Ω·cm^2^). Besides, the TEER of the Caco-2 model was in accordance with Briske-Anderson et al., who cultured the Caco-2 cells from passage 22 with similar conditions and obtained a TEER value at around 500 Ω·cm^2^ after 21 days [43,44,45,46,47,48,49].

**Figure 3:**
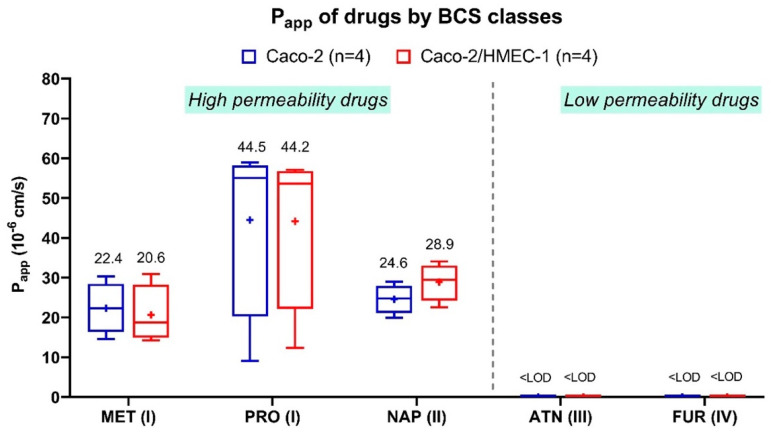
Average apparent permeability (P_app_) of five drugs (*n* = 4) classified by four BCS classes across Caco-2 cells (blue) and HMEC-1/Caco-2 coculture cells (red). MET = metoprolol, PRO = propranolol, NAP = naproxen, ATN = atenolol, FUR = furosemide; BCS class numbers are signified in the parentheses. LOD means the limit of detection = 10 ng/mL. The (+) symbol represents the arithmetic mean, and the whiskers represent a 95% confidence interval (Kruskal–Wallis).

Since this new in vitro coculture model was intended as a tool to assess drug absorption, the apical-to-basolateral P_app_ of five reference drugs from all four BCS classes was evaluated and compared with their P_app_ from the conventional Caco-2 model (Figure 3). The five drugs in this experiment are chosen because they are commonly used as the reference for evaluating the permeability in the Caco-2 model. Their solubility and permeability are defined by the BCS [45,46,47,48,49,50,51]. For atenolol (class III) and furosemide (class IV), the concentration at the basolateral side was lower than the detection limit in both coculture and Caco-2 models. These results were in accordance with the definition of classes III and IV, which have low apparent permeability. For propranolol (class I), metoprolol (class I), and naproxen (class II), their P_app_ were ranging from 20.6 to 44.2 × 10^−6^ cm/s on the coculture model and 22.4 to 44.5 × 10^−6^ cm/s on the Caco-2 model, conforming with the BCS classification as well. No significant differences (Kruskal–Wallis) between the P_app_ of all five drugs across the coculture and the Caco-2 model were obtained. Therefore, the P_app_ of both Caco-2/HMEC-1 and Caco-2 models were in the same range for the five tested drugs. These values were also similar to those from various literature that high-permeability drugs in BCS class I and II had the P_app_ in the range from 9 × 10^−6^ to 43 × 10^−6^ cm/s, while low-permeability drugs in class III and IV had nearly zero P_app_ [6,7,10,11,12,13,14,15,16,17]. In addition, the P_app_ of the drugs across the HMEC-1 monolayer and the blank Transwell^®^ filter (Figure S1) was also examined as a control. The P_app_ of all drugs were in the same range from 22 × 10^−6^ to 34 × 10^−6^ cm/s with no significant difference (Kruskal–Wallis) between drugs of high and low permeability across both HMEC-1 monolayer and blank filter. In conclusion, the addition of the HMEC-1 layer in the coculture model did not change the permeability of these five references drugs across the membranes, suggesting that the conventional Caco-2 model alone might be adequate for studying the absorption of drug molecules.

### 3.2. Transport Assay of Intact LNCs across Membranes

LNCs demonstrated their ability to improve the oral absorption of several encapsulated drugs [50]. Nanoparticle size can also influence oral drug absorption [7,51]. Furthermore, surface-modified LNCs such as PEG (2000)-amino post-inserted LNCs and stearylamine LNCs can enhance oral drug bioavailability [21]. Hence, in order to study the impact of size and surface chemistry of LNCs on their oral in vitro absorption, the LNCs with two different sizes (F1 and F2) and three different surface chemistry (unmodified LNCs, anionic DSPE-mPEG (2000) added, and cationic stearylamine added) were formulated (Table 2) and tested in both coculture and Caco-2 models. In addition, Roger et al. have recently shown that the FRET technique could be used to detect and quantify the intact LNCs crossing the Caco-2 monolayer [4,5]. Thus, in order to monitor the integrity of LNCs across the membrane, the FRET technique was used.

Table 3 presents the physicochemical characteristics of the FRET-LNCs in terms of hydrodynamic size, particle size distribution, particle concentration, zeta potential, and FRET proximity ratio. The size distribution histogram is shown in Figure S2. The composition F1 and F2 provided the FRET-LNCs with an average diameter of around 55 nm and 85 nm, respectively. The F2 formulation group has a higher amount of Captex^®^ 8000 (oily core) and a slightly lower amount of Kolliphor^®^ HS-15 than the F1 group. In accordance with the ternary diagram of a mixture system established by Heurtault et al. [6,10] and other previous studies [5], increasing the amount of oil composition together with decreasing the amount of the hydrophilic surfactant (Kolliphor^®^ HS-15) could increase the size of LNCs [21]. Moreover, the particle size distribution of all formulations was unimodal and uniform (Figure S2). The D50 value is the median diameter (nm). All the formulations had a symmetric distribution as their median diameter was almost equal to the mean diameter. Besides, span is the parameter determining the size distribution width normalized by median diameter. The span value closing to zero determines a narrow size distribution. The span of the compositions F1 (0.37–0.38) was lower than the F2 (0.54–0.63), indicating that LNCs with smaller sizes had a narrower distribution width, while the surface modification had no effect on altering the distribution width (Kruskal–Wallis). Furthermore, both F1 and F2 compositions had similar particle concentrations (Kruskal–Wallis) ranging from 5.5 × 10^14^ to 8.0 × 10^14^ particles/mL and slightly lower than previously reported in the literature at 1.2 × 10^15^ particles/mL for 55 nm LNCs [20]. By contrast, the zeta potential of F2 was higher than F1 (16.3 ± 3.7 mV and 4.1 ± 0.8, respectively), but such a difference was not observed in the blank F1 and F2 formulations (containing no FRET dyes), of which the zeta potential was around −5.0 mV regardless of sizes. The FRET dyes DiI-TPB and DiD-TPB used in the formulation were positively charged and were previously reported to elevate the zeta potential of 55-nm LNCs [6]. The fact that F2 had a quantity of Captex^®^ 8000, in which FRET dyes were dissolved, higher than F1 could explain the higher zeta potential in F2.

Adding anionic DSPE-mPEG (2000) and cationic stearylamine at the formulation of FRET-LNCs did not significantly change the size and the particle concentration (Kruskal–Wallis, see Table 3). However, adding DSPE-mPEG (2000) decreased the zeta potential by 9–13 mV, whilst stearylamine increased it by 11 mV, as already described by Lainé et al. [7] and Ramadan et al. [52,53].

Finally, to determine the integrity of LNCs, the FRET proximity ratio (PR) was calculated [21]. Intact LNCs have PR closer to 1 due to the highly efficient energy transfer between FRET dyes in close proximity, while broken LNCs can have PR as low as 0.22 or less [54]. The PR of all formulations (Table 3) ranged from 0.89 to 0.93, meaning that the FRET dyes DiI-TPB/DiD-TPB were well encapsulated, and the formulations were full of intact LNCs. Size or surface modifications had no significant effect on PR. In summary, six FRET-LNCs formulations with different sizes and surface modifications were successfully developed and suitable for the transport assay experiment.

**Figure 4:**
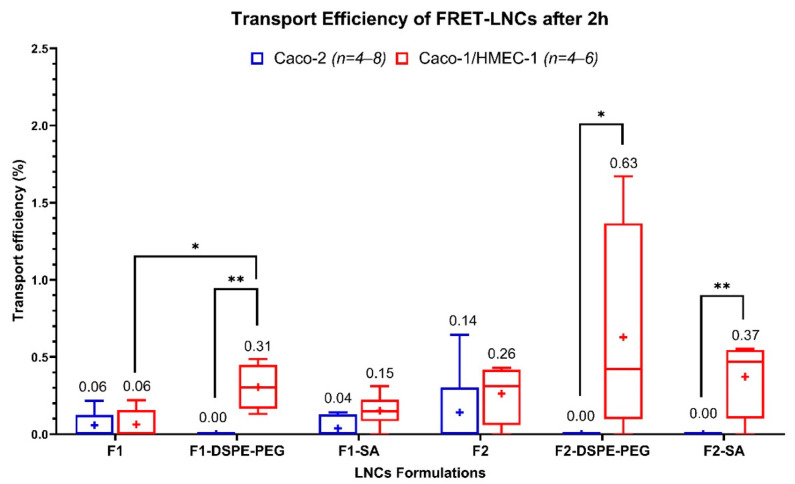
Transport efficiency of six FRET-LNCs formulations after 2 h in the Caco-2 model (blue, *n* = 4–8), and the Caco-2/HMEC-1 coculture model (red, *n* = 4–6). The (+) symbols represent the arithmetic mean, and the whiskers represent a 95% confidence interval (Kruskal–Wallis: * *p* ≤ 0.05, ** *p* ≤ 0.01).

The transport of the six formulations was investigated in the Caco-2 and the Caco-2/HMEC-1 coculture model. The transport efficiency (TE) of each formulation is shown in Figure 4. In the Caco-2 model, when compared by sizes and surface-chemistry, the TEs of all different LNCs did not show statistically significant differences (Kruskal–Wallis). In the coculture model, when compared by sizes, the F2, F2-DSPE-PEG, and F2-SA had higher trends of TEs than their F1 counterparts, but no statistical significance (Kruskal–Wallis) was found. Furthermore, when compared by surface-chemistry, only F1-DSPE-PEG could increase the TEs of F1 with a statistical significance (Kruskal–Wallis). Finally, when compared between models, the TEs of F1-DSPE-PEG, F2-DSPE-PEG, and F2-SA were found to be higher, with a statistical significance (Kruskal–Wallis), in the coculture model than in the Caco-2 model, while in the coculture model, the TEs of F1-DSPE-PEG, F2-DSPE-PEG, and F2-SA were lower than the detection limit (reported as TE = 0). Summarily, results clearly showed a different pattern of TEs between the two models, meaning that the addition of an endothelium layer to the Caco-2 one increases the TE of F1-DSPE-PEG, F2-DSPE-PEG, and F2-SA. LNCs size did not affect TE in both models, while the surface chemistry had an effect on TE but only with F1-DSPE-PEG (55-nm).

Using the new coculture model revealed that F1-DSPE-PEG had a significantly higher transport efficiency than F1, meaning that adding DSPE-mPEG (2000) to the surface of 55-nm LNCs increased their in vitro intestinal absorption. A similar result has been observed by Bannunah et al. [54], who described that anionic polystyrene nanoparticles (PS-NPs) had much higher transportation across the Caco-2 model than cationic PS-NPs. More precisely, transcytosis of anionic PS-NPs mainly occurred via the caveolae-mediated pathway, while it was not the case for cationic PS-NPs whose transcytosis occurred via the clathrin-mediated pathway [55,56]. Moreover, adding more PEG chains, like DSPE-mPEG (2000), to the surface of LNCs would also improve their mucopenetrating property [43,44,45,46,47,48].

Interestingly, the different absorption patterns between the two models only appeared among the LNCs but not among the reference molecular drugs (Figure 3). Some LNCs, but none of the reference drugs, had significantly higher absorption in the coculture model despite its higher membrane integrity (TEER). Molecular drugs are mainly absorbed via the passive pathway regulated by the membrane’s chemical properties rather than the biological ones [10,57,58,59]. By contrast, the LNCs are absorbed by active transport via the caveolae-mediated or clathrin/caveolae-independent endocytosis in the Caco-2 model [57,58,59]. The hydrophilic surfactant (Kolliphor^®^ HS-15, previously named Solutol^®^ HS-15) on the outer shell of LNCs could directly interact with the cholesterol-rich microdomain (lipid raft) on the cell surface, inciting the endocytosis [57,58,59]. The difference in LNCs absorption between the Caco-2 and the coculture models implied that adding the HMEC-1 layer might cause a change in the cell’s biological absorption process that affected the LNCs transcytosis, but not the membrane’s chemical properties that affected molecular drugs absorption. Therefore, the next step of this work is to determine the LNCs transport mechanism across this coculture model.

## 4. Conclusions

In conclusion, a new coculture model of Caco-2 intestinal epithelium and human primary vascular endothelium cells (HMEC-1) was successfully developed. The cell morphology, membrane integrity, and drug permeability were validated and proved that the new model was suitable for a tool to evaluate the absorption of drugs and nanoparticles. Improving the conventional Caco-2 model by adding the endothelium layer could change the absorption pattern of LNCs but not drugs in solution. This suggests the necessity of the new coculture model for studying the absorption of LNCs or other nanocarrier systems. However, for studying the absorption of drug in solutions, the Caco-2 model should be adequate. By using the coculture model, the absorption of DSPE-mPEG (2000) LNCs (55- nm and 85-nm) and stearylamine LNCs (85-nm) was found to be surprisingly higher, despite the coculture model’s higher TEER. Furthermore, the new model also revealed the increase in the absorption of 55-nm LNCs when surfaced-modified by DSPE-mPEG (2000). All these distinct absorption patterns between the two models imply the differences in the LNCs transport mechanism and are the proof of concept for the effect and the importance of the intestinal endothelium on the transportation of nanoparticles across gut barriers. The use of the new coculture model combined with the FRET technique could provide a better understanding of the fate of intact LNCs across the intestinal barriers. For future experiments, the details on the transport mechanism of LNCs in the coculture model should be elucidated. The in vivo–in vitro correlation (IVIVC) of intact LNCs should be further investigated. Besides, the new model should also be used to study the absorption of other types of nanocarriers.

## Figures and Tables

**Table 1:** Classification of the studied drugs according to the BCS.

BCS Classes	Solubility	Permeability	Drugs
I	High	High	Metoprolol tartrate, Propranolol HCl
II	Low	High	Naproxen
III	High	Low	Atenolol
IV	Low	Low	Furosemide

**Table 2:** Composition of different types of FRET-LNCs.

Compositions	Quantity (% *w*/*w*)					
	F1	F1-DSPE-PEG	F1-SA	F2	F2-DSPE-PEG	F2-SA
Captex^®^ 8000 (2% *w*/*w* DiI-TPB)	5.5	5.5	5.5	8.5	8.5	8.5
Captex^®^ 8000 (2% *w*/*w* DiD-TPB)	5.5	5.5	5.5	8.5	8.5	8.5
Kolliphor^®^ HS-15	11.5	11.5	11.5	9.3	9.3	9.3
Purified water	21.3	21.3	21.3	17.5	17.5	17.5
DSPE-mPEG (2000)	-	0.6	-	-	0.6	-
Stearylamine	-	-	0.1	-	-	0.1
Lipoid^®^ S75-3	0.7	0.7	0.7	0.7	0.7	0.7
NaCl	0.8	0.8	0.8	0.8	0.8	0.8
Purified water (2 °C)	54.7	54.7	54.7	54.7	54.7	54.7

**Table 3:** Characterization of FRET-LNCs (mean ± SD): particle size, particle size distribution, particle concentration, zeta potential, and proximity ratio.

Formulas	Particle Size (nm)	Particle Size Distribution				Particle Concentration (×10^14^ Particles/mL)	Zeta Potential (mV)	FRET Proximity Ratio
		D10 (nm)	D50 (nm)	D90 (nm)	Span			
F1 (*n* = 3)	57.8 ± 9.7	45.3 ± 5.0	54.2 ± 7.7	65.6 ± 12.5	0.37 ± 0.08	7.7 ± 1.2	4.1 ± 0.8	0.89 ± 0.04
F1-DSPE-PEG (*n* = 2)	53.1	42.7	50.9	62.5	0.38	7.9	−4.9	0.89
F1-SA (*n* = 3)	56.0 ± 5.6	45.3 ± 4.5	53.5 ± 5.1	65.6 ± 7.2	0.38 ± 0.04	8.0 ± 3.1	15.4 ± 2.1	0.89 ± 0.04
F2 (*n* = 3)	92.6 ± 10.0	67.1 ± 6.2	88.5 ± 8.9	120.4 ± 15.3	0.60 ± 0.04	5.5 ± 2.7	16.3 ± 3.7	0.93 ± 0.02
F2-DSPE-PEG (*n* = 2)	83.3	61.3	77.3	109.9	0.63	6.6	3.2	0.92
F2-SA (*n* = 3)	82.6 ± 5.8	62.3 ± 3.7	78.7 ± 5.2	104.7 ± 9.9	0.54 ± 0.07	6.8 ± 2.3	27.3 ± 2.8	0.93 ± 0.02

## Data Availability

All data generated or analyzed during this study are included in this published article and its Supplementary information files.

## Supplementary Materials

The following are available online at https://www.mdpi.com/article/10.3390/pharmaceutics13050595/s1, Figure S1: Average apparent permeability (P_app_) of five drugs (*n* = 4) classified by four BCS classes across HMEC-1 monolayer (green) and blank Transwell^®^ filter (gray). MET = metoprolol, PRO = propranolol, NAP = naproxen, ATN = atenolol, FUR = furosemide; BCS class numbers are signified in the parentheses. The (+) symbol represents the arithmetic mean, and the whiskers represent a 95% confidence interval (Kruskal-Wallis). Figure S2: Example of the particle size distribution histogram of the formulations (A–F): F1, F1-DSPE-PEG, F1-SA, F2, F2-DSPE-PEG, and F2-SA, respectively.

## Appendix A. Sample Preparation for Drug Assay by HPLC-UV

### Appendix A.1. Sample Preparation for Metoprolol and Propranolol

The internal standard was prepared as a solution of 20 mg/L protriptyline (in methanol) for propranolol and 20 mg/L methyl milnacipran (in methanol) for metoprolol. Then, 25 µL of the internal standard and 100 µL of 4 M NaOH were added to 500 µL of the sample. Afterward, liquid-liquid extraction using 4 mL of hexane/isoamyl alcohol (80/20, *v*/*v*) was performed, and 100 µL of 0.02 M HCl was added to the organic phase after mixing. The mixture was centrifugated, and the supernatant was eliminated. 10 µL of the preparation was injected into the chromatographic system.

### Appendix A.2. Sample Preparation for Naproxen

The internal standard was prepared as a solution of 50 mg/L tolbutamide (in acetonitrile). Then, 200 µL of the internal standard was added to 50 µL of the sample. The preparation was then centrifuged, and 10 µL of the supernatant was injected into the chromatographic system.

### Appendix A.3. Sample Preparation for Atenolol

The internal standard was prepared as a solution of 20 mg/L prazepam (in methanol). Then, 25 µL of the internal standard was added to 500 µL of the sample. Afterward, liquid-liquid extraction using 5 mL of dichloromethane and 30 µL of 1 M NaOH was performed. After mixing, the mixture was centrifugated, and the supernatant (organic phase) was collected and evaporated to dryness at 50 °C under nitrogen gas. The residue was dissolved in 50 µL of methanol and 20 µL of water. 10 µL of the preparation was injected into the chromatographic system.

### Appendix A.4. Sample Preparation for Furosemide

The internal standard was prepared as a solution of 50 mg/L tolbutamide (in acetonitrile). Then, 25 µL of the internal standard and 200 µL of ammonium acetate buffer pH 4.0 were added to 200 µL of the sample. Afterward, liquid-liquid extraction using 1.5 mL of dichloromethane was performed. After mixing, the mixture was centrifugated, and the supernatant (organic phase) was collected and evaporated to dryness at 50 °C under a nitrogen stream. The residue was dissolved in 50 µL of methanol and 20 µL of water. 10 µL of the preparation was injected into the chromatographic system.
