# Supplementary material for: New In Vitro Coculture Model for Evaluating Intestinal Absorption of Different Lipid Nanocapsules
Source: Pharmaceutics. 2021 Apr 21;13(5):595. doi: 10.3390/pharmaceutics13050595 (PMC8143299; doi:10.3390/pharmaceutics13050595)
Supplement: Supplementary file 1 [file pharmaceutics-13-00595-s001.zip › pharmaceutics-1192884-supplementary.pdf]

pharmaceutics

Supplementary Materials: New In Vitro Coculture Model for Evaluating Intestinal Absorption of Different LipidNanocapsules

Norraseth Kaeokhamloed, Emillie Roger, Jérôme Béjaud, Nolwenn Lautram, Florence Manero, Rodolphe Perrot, Chadi Abbara, Marie Briet and Samuel Legeay

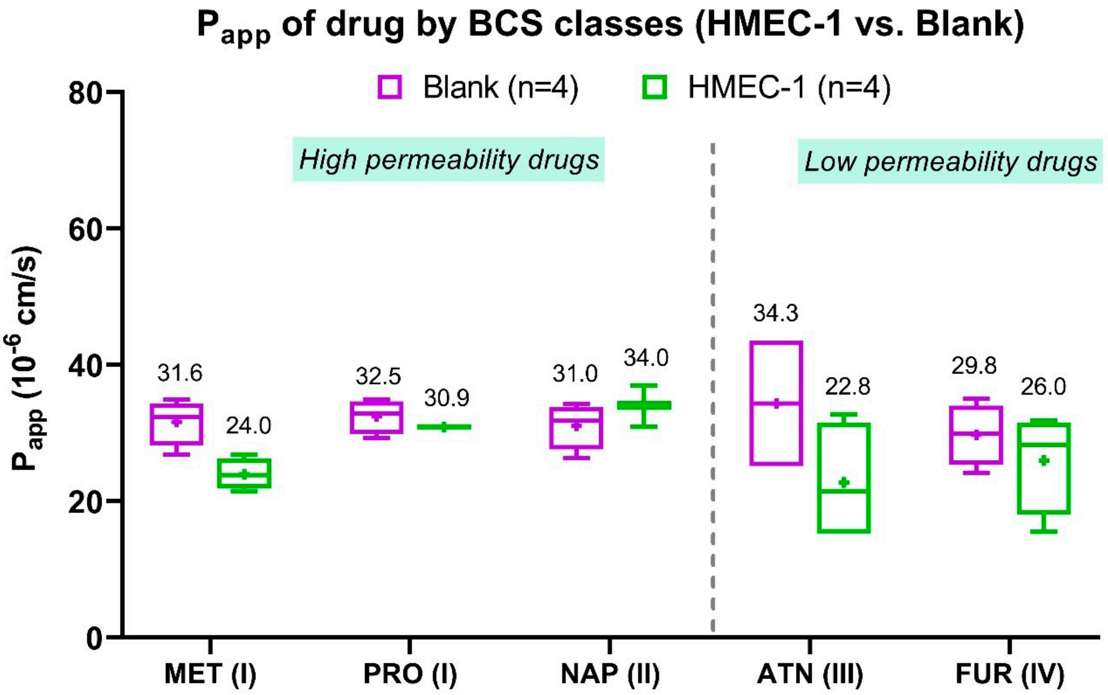

Figure S1. Average apparent permeability (P_{app}) of five drugs (n = 4) classified by four BCS classes across HMEC-1 monolayer (green) and blank Transwell® filter (gray). MET = metoprolol, PRO = propranolol, NAP = naproxen, ATN = atenolol, FUR = furosemide; BCS class numbers are signified in the parentheses. The (+) symbol represents the arithmetic mean, and the whiskers represent a 95% confidence interval (Kruskal-Wallis).

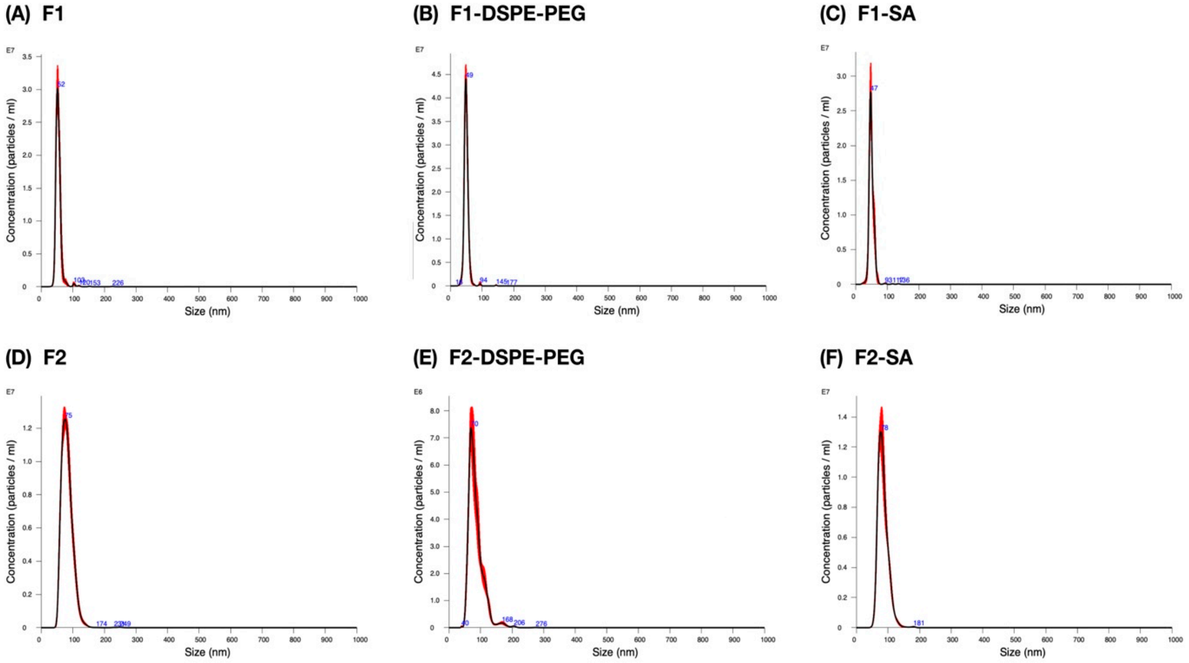

Figure S2. Example of the particle size distribution histogram of the formulations (A–F): F1, F1-DSPE-PEG, F1-SA, F2, F2-DSPE-PEG, and F2-SA, respectively.
